# Severe Hydronephrosis and Dysuria-Hematuria Syndrome after 20 Years of Bladder Exstrophy Correction: A Case Report

**DOI:** 10.1155/2012/324510

**Published:** 2012-11-11

**Authors:** Emanuela Altobelli, Alfredo Maria Bove, Federico Sergi, Marzio Angelo Zullo, Maurizio Buscarini

**Affiliations:** ^1^Urology Department, Universitá Campus Bio-Medico di Roma, Via Alvaro del Portillo 200, 00128 Rome, Italy; ^2^Gynecology Department, Universitá Campus Bio-Medico di Roma, Via Alvaro del Portillo 200, 00128 Rome, Italy

## Abstract

The aim of this paper is to report a case of severe hydronephrosis and incontinence 20 years after bladder exstrophy repair, managed successfully by secondary ureteroneocystostomy and by transurethral submucosal injection of Macroplastique.

## 1. Introduction

Bladder exstrophy (BE) is an extremely rare congenital abnormality that belongs to the wide spectrum of the epispadias-exstrophy complex, with an incidence of 2.15 per 100,000 live births. The male-to-female ratio is almost even. White infants are significantly more likely to present with exstrophy than nonwhites [1]. All BE patients develop vesicoureteral reflux (VUR). Hydronephrosis following ureteral reimplantation (UR) is common occurrence. In most cases, postoperative hydronephrosis is transient and not clinically significant, with a high incidence of complete resolution during the first 2 years. Ureteral obstruction occurs rarely following reimplantation.

On the other hand, urinary incontinence continues to be one of the most challenging problems in patients with BE. The primary management of BE has developed over the last few decades. The surgical treatment of females with BE has included reconstruction of the external genitalia at the time of the initial closure. Bladder neck reconstruction is typically deferred until continence is desired and generally not needed in female patients. Achieving continence is a major long-term goal of urological management in these patients.

We report a case of severe hydronephrosis associated to febrile urinary tract infections (UTIs) 20 years after bladder exstrophy repair, managed successfully by bilateral ureteral tapering and secondary ureteroneocystostomy and followed after 2 months by new onset incontinence, managed successfully by transurethral submucosal injection of Macroplastique.

## 2. Case Presentation

A 21-year-old female patient with a history of bladder exstrophy (BE) was referred to our department for dysuria-hematuria syndrome and recurrent febrile urinary tract infections (UTIs).

Her surgical history started on the fourth day of life. During the first procedure, she underwent bladder closure without osteotomy in a staged reconstructive approach. At 6 years for low bladder capacity, a gastrocystoplasty was performed to correct low bladder capacity. At the age of 10, she underwent a bilateral ureteral reimplantation (UR) according to Cohen for hydronephrosis secondary to vesicoureteral reflux (VUR) due to high bladder pressure. The last procedure was performed at 19 years with the removal of umbilical endometriosis.

**Figure 1:**
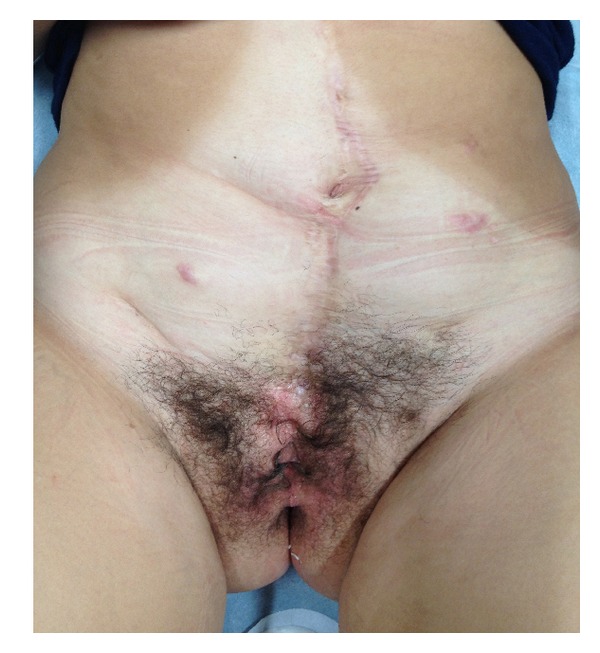
Abdomen examination.

On physical examination (Figure 1), she presented with lower midline and Pfannenstiel scars due to previous procedures, pubis diastasis, bifid clitoris, stenotic and anteriorly displaced vagina, and short perineum with the anus directly behind the urogenital diaphragm. No alterations on blood tests, with normal renal function (creatinine 0.7 mg/dL). Urine culture showed >100.000 CFU of *E. coli* sensitive to ceftriaxone. Antibiotic was prescribed for 7 days. To treat the dysuria-hematuria syndrome, a proton pomp inhibitor (PPI) therapy was started.

**Figure 2:**
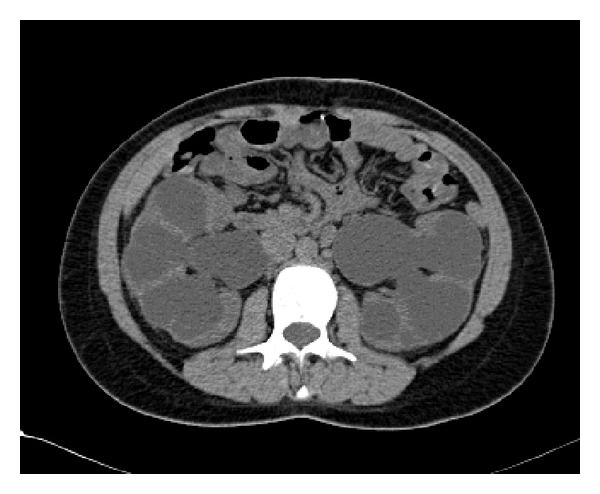
Preoperative CT scan.

A severe bilateral hydronephrosis, greater on the left, associated to a compression of renal parenchyma, was apparent at ultrasonography (US) and was confirmed at the CT scan (Figure 2), with an AP pelvic diameter of 33 mm on the right and 43 mm on the left. Right and left ureters presented diameters of 23 and 40 mm, respectively. A high postvoid residual volume was evident.

The patient refused self-intermittent clean catheterisation. She allowed us to perform cystourethroscopy under general anaesthesia; this showed a long and twisting urethra with a bladder full of mucus without alterations of urothelial and gastric mucosa. Cross trigonal ureteral meatus were stenotic bilaterally, and retrograde stenting was not possible. During the same procedure, a retrograde cystourethrogram was executed without evidence of VUR. One month later, she elected to undergo open surgery.

## 3. Surgical Technique

Because of previous surgical procedures, a laparoscopic approach was not indicated. A 18 Fr transurethral foley catheter was inserted. Patient was placed in supine position with 15° Trendelenburg tilt. After sterile draping, a lower midline incision was performed. After peritoneal incision, the exposure was obtained with a self-retaining retractor. The bulging and dilated ureters were identified extravesically and placed on a gentle traction with a vessel loop. Ureters were mobilized to the ureterovesical junction, and a stiff and severe anastomotic stricture determining obstruction was verified bilaterally. Ureters were divided and hiatus oversewn with 4-0 Polyglactin suture. A 4-0 Polyglactin stay suture was placed on the distal end of each ureter. Both ureters were fully mobilized up to the crossing of the common iliac vessels. Once the ureters were mobilized and decompressed, a decision was made to proceed with extensive ureteral tailoring and reimplant. To achieve a tunnel length-to-diameter ratio of at least 5 : 1, a 10 Fr catheter was placed in the lumen of each ureter, and these were tapered using Hendren clamps. Only the intramural part of the ureter was tapered. The bladder was opened via a midline cystotomy and the ureter was reimplanted with an antireflux intravesical anastomosis according to Glenn-Anderson technique on left ureter. Right ureter was reimplanted with Lich-Gregoir extravesical approach. A mono-J ureteral catheter was retrograde-positioned in both ureters. The bladder was closed in two layers using 3-0 Polyglactin. A perivesical Jackson-Pratt drain was left in place.

## 4. Results

Postoperatively, the patient was allowed to resume a regular diet. Drain and Foley catheters were removed on days 3 and 6, respectively. Cystoscopy and cystourethrogram were performed as an outpatient 4 weeks postoperatively to remove ureteral stents and transurethral catheter. Urine culture 1 month after surgery was positive for *E. coli* growth, and antibiotic therapy was prescribed based on urine culture results.

Two months after the procedure, she complained about incontinence during orthostatism and deambulation using about 12 pads/day, with dry intervals <3 hours/day. She underwent physiotherapy with Kegel's pelvic floor exercises for 2 months with slight improvement, so she started daily sessions of pelvic floor electrostimulation for other 2 months without benefit. Patient refused urodynamic evaluation because of childhood catheter trauma; so to investigate the symptoms we decided to perform under general anaesthesia a cystourethroscopy that showed a long urethra with an open bladder neck and no alterations of urothelial and gastric mucosa. Ectopic and ejaculant ureteral meatus were identified bilaterally. Sling insertion was not indicated because of short perineum. Two weeks later, she elected to undergo operative cystoscopy.

## 5. Surgical Technique

**Figure 3:**
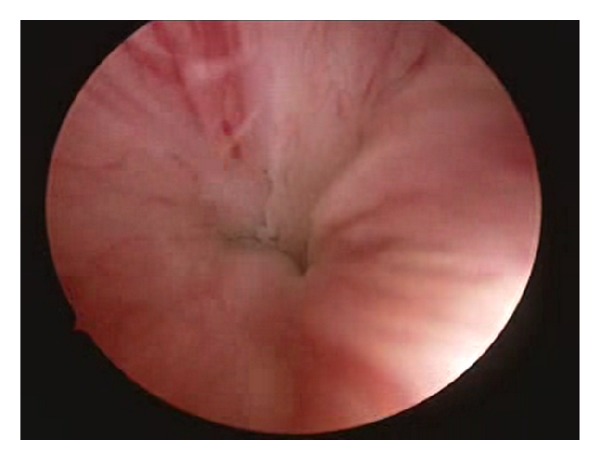
Coapted bladder neck.

Eight months after ureteral reimplantation, to treat the incontinence, she was scheduled for transurethral submucosal injection of Macroplastique. Informed consent was obtained from the patient. Urine was sterile at the time of treatment. Under general anaesthesia, patient was placed in a lithotomy position. During cystoscopy open bladder neck was evident. A combined antegrade-retrograde approach was used, the needle was placed submucosally and under direct vision a bleb of 2.5 mL was raised at 6 o'clock site and two other blebs of 1.25 mL at 2 and 10 o'clock sites, for a total volume of 5 mL. A coapted bladder neck was visualized at the end of the procedure (Figure 3).

## 6. Results

No perioperative complications were recorded. No clean intermitted catheterization was needed. 1, 3, and 6 months after surgery, she reported complete continence, with occasional use of 1 pad/day, with a dry interval >6 hours/day. Blood tests presented a normal renal function (creatinine 0.7 mg/dL) and urine culture was negative for bacterial growth. Patient was asymptomatic. Quality of life was greatly improved.

## 7. Discussion

After initial closure, all exstrophy patients have vesicoureteral reflux. In bladder exstrophy, the ureters dip deeply into the pelvis before entering the bladder in a nearly cephalad direction, emerging through the bladder muscle with a minimal submucosal tunnel [2]. This reflux is usually managed with antimicrobial prophylaxis, surveillance, and ureteral reimplantation concurrent with bladder neck plasty [3–6]. Our patient developed recurrent urinary tract infections and worsening hydronephrosis, and at the age of 10 underwent first bilateral UR according to Cohen's technique. During the procedure bladder neck plasty was not performed.

However, ureteral reimplantation is effective in reducing postoperative pyelonephritis (POP) [7], UTI is the most common complication. There is surprisingly little literature describing the long-term clinical outcomes after UR. The International Reflux Study in Children (IRSC) prospectively compared the effects of successful surgery and effective medical management on the incidence of UTIs. During a followup of 5 years, that study reported an incidence of 38% of infections of the lower urinary tract among conservatively treated patients, compared with 39% among those treated by surgery. The incidence of pyelonephritis was 21% and 10%, respectively, and was statistically significantly different [8]. In another long-term, retrospective study of patients 10 years after UR, Beetz et al. found that, among the 83.5% of patients they could contact, 17% had experienced postoperative febrile UTI [9]. In contrast to these high rates of POP, Whittam et al. reported in a recent series that of 395 patients who underwent UR febrile UTI were diagnosed in just 4.6%, although the follow-up time was relatively short at a mean of 15 months [10]. Accordingly, Cooper and Atwell reported an incidence of 37.6% of UTIs among 96 women who had ureteric reimplantation in childhood and who were followed for 16–25 years afterward [11]. As emphasized by Mor et al., there is a need to establish a protocol for the long-term followup of patients who have had ureteric reimplantation during childhood. Even patients who were managed successfully by surgery are prone to recurrent UTIs, progressive renal scarring, hypertension, and complications during pregnancy [12]. Our patient present only recurrent febrile UTIs associated to severe hydronephrosis. The primary complication that is assessed by postoperative imaging is obstruction and presents clinically in addition to ultrasonographically [13–15]. Ellsworth et al. reported on 3 cases of ureteral obstruction during the first week after surgery. All of these patients were symptomatic and were treated with the placement of a double-J stent [16]. Androulakakis et al. also reported 3 incidences of obstruction, all of which presented symptomatically, and only one of which required reoperation [17]. Additionally, temporary obstructive situations may occur following surgery, including ureteral edema, intramural hematomas applying pressure on the ureter, temporary lack of peristalsis in the operated ureter, and kinking of the ureter at the hiatus as the bladder fills [18]. These obstructions can be partial and self-limiting following surgery, are varied between each patient, and contribute to the length of time it takes for postoperative hydronephrosis to resolve. They do not represent clinically important obstruction however [19–21]. All these studies evaluate only a short-term followup. There are no studies about worsening hydronephrosis 20 years after UR.

The other major complication of BE is incontinence. To treat incontinence in patients with BE, the achievement of sufficient outlet resistance and a compliant nonoveractive bladder with adequate capacity are necessary. Bladder outlet resistance can be increased by major surgical procedures, such as bladder neck reconstruction (BDR), slings, urethral lengthening, or an artificial urinary sphincter. However, these procedures do not reliably achieve a satisfactory result in every patient [22–26]. On the other hand, in many instances the cost of dryness is the loss of the ability to void spontaneously and patient must perform clean intermitted catheterization (CIC) to empty the bladder. In extreme cases, when previous procedures have failed, the bladder neck must be closed and continent catheterizable reservoir created [27, 28]. Our patient was not incontinent prior to last surgery, her continence was probably false and due to the long and twisting urethra that became straight during steady filling of the bladder. This simulated continence was associated to high postvoid volume that caused febrile UTIs and hydronephrosis and necessity of open surgery, in which a transurethral Foley catheter was inserted. After catheter removing worsening incontinence started to compare; we decided to perform an endoscopic correction of the incontinence. Endoscopic treatment of urinary incontinence due to bladder outlet deficiency has gained increasing acceptance in recent years because it is minimally invasive and takes immediate effects. In the mid-1970s, Politano first described Teflon to treat incontinence [29]. In 1985, Vorstman et al. reported on 11 patients, of whom 8 were injected retrogradely and 5 were injected perineally, with a followup of 5.3 years [30]. Of that cohort, 45% were considered dry and an additional 27% were considered improved at last followup. However, most bulking agents used in the early phase, such as polytetrafluoroethylene paste, or bovine collagen, have been abandoned due to safety concerns. Thus, the only polymethylsiloxane (Macroplastique) or dextranomer based implants (Deflux) have recently been used for this indication. Regarding short-term results, Halachmi et al. used Macroplastique and noted 42% improved continence in 28 children in a 13-month study in 2004, although none achieved complete dryness [31]. In 2003 in a 5-year follow-up study of Macroplastique, 3 of 15 children were reported to be cured [32]. In the series by Caione and Capozza, 19 patients, of whom 11 previously underwent bladder neck surgery, were treated via a retrograde approach with an average of 2.8 cc Deflux. Patients required between 1 and 3 injections. A total of 13 patients had exstrophy/epispadias and 3 had a congenitally neuropathic bladder. At 1 year of followup, 56.3% of patients showed improvement and 18.7% were cured, defined as 2.5 hours of continence [33]. Most of these series included patients with previous BNR, our patient had previous pelvic surgery but not BNR, and anyway continence was achieved. Positive aspects in favour of bulking agents are lower costs, shorter hospitalization, and less trauma in the patient than major continence surgical procedures [34]. Interestingly, Guys et al. reported the results of a similar prospective study in 46 children with major structural incontinence related to neuropathic bladder dysfunction that was treated with endoscopic polydimethylsiloxane injections [35]. On the other hand, Dyer et al. in their series of 34 patients who underwent primary injection of bulking agents did not find a significant improvement in continence, concluding that is often ineffective and expensive [36]. In 2006, Burki et al. presented long-term results of Macroplastique injection in 52 children with exstrophy-epispadias complex, with a median followup of 4.6 years, confirming that injection with Macroplastique is significantly durable in many patients with a reasonable success rate [37].

## 8. Conclusions

Even if there are some studies about the postoperative UTIs and hydronephrosis, there are no data about reintervention 20 years after primary UR. Our case demonstrates the success of UR associated to ureteral tapering even after a prior reimplantation in bladder exstrophy patient. Macroplastique injection is safe, minimally invasive, and successful in the treatment of incontinence in patient with previous BE, even as a primary treatment.

## References

[1] Nelson CP, Dunn RL, Wei JT (2005). Contemporary epidemiology of bladder exstrophy in the United States. *Journal of Urology*.

[2] Canning DA, Gearhart JP, Peppas DS, Jeffs RD (1993). The cephalotrigonal reimplant in bladder neck reconstruction for patients with exstrophy or epispadias. *Journal of Urology*.

[3] Grady RW, Mitchell ME (1999). Complete primary repair of exstrophy. *Journal of Urology*.

[4] Nisonson I, Lattimer JK (1972). How well can the exstrophied bladder work?. *Journal of Urology*.

[5] Baker LA, Gearhart JP (1998). The staged approach to bladder exstrophy closure and the role of osteotomies. *World Journal of Urology*.

[6] Mathews R, Hubbard JS, Gearhart JP (2003). Ureteral reimplantation before bladder neck plasty in the reconstruction of bladder exstrophy: indications and outcomes. *Urology*.

[7] Nelson CP, Hubert KC, Kokorowski PJ Long-term incidence of urinary tract infection after ureteral reimplantation for primary vesicoureteral reflux.

[8] Jodal U, Koskimies O, Hanson E (1992). Infection pattern in children with vesicoureteral reflux randomly allocated to operation or long-term antibacterial prophylaxis. *Journal of Urology*.

[9] Beetz R, Mannhardt W, Fisch M, Stein R, Thuroff JW (2002). Long-term followup of 158 young adults surgically treated for vesicoureteral reflux in childhood: the ongoing risk of urinary tract infections. *Journal of Urology*.

[10] Whittam BM, Thomasch JR, Makari JH (2010). Febrile urinary tract infection after ureteroneocystostomy: a contemporary assessment at a single institution. *Journal of Urology*.

[11] Cooper A, Atwell J (1993). A long-term follow-up of surgically treated vesicoureteric reflux in girls. *Journal of Pediatric Surgery*.

[12] Mor Y, Leibovitch I, Zalts R, Lotan D, Jonas P, Ramon J (2003). Analysis of the long-term outcome of surgically corrected vesico-ureteric reflux. *BJU International*.

[13] El Imam Mohammed M, Omram M, Nugud F (2005). Evaluation of ureteral reimplantation in 65 Sudanese patients. *Saudi Journal of Kidney Diseases and Transplantation*.

[14] Jodal U, Smellie JM, Lax H, Hoyer PF (2006). Ten-year results of randomized treatment of children with severe vesicoureteral reflux. Final report of the International Reflux Study in Children. *Pediatric Nephrology*.

[15] Kennelly MJ, Bloom DA, Ritchey ML, Panzl AC (1995). Outcome analysis of bilateral cohen cross-trigonal ureteroneocystostomy. *Urology*.

[16] Ellsworth PI, Freilich DA, Lahey S (2008). Cohen cross-trigonal ureteral reimplantation: is a one-year post-operative renal ultrasound scan necessary after normal initial post-operative ultrasound findings?. *Urology*.

[17] Androulakakis PA, Stefanidis AA, Karamanolakis DK, Moutzouris V, Koussidis G (2003). The long-term outcome of bilateral Cohen ureteric reimplantation under a common submucosal tunnel. *BJU International*.

[18] Khoury AE, Darius JB (2011). *Vesico-Ureteral Reflux*.

[19] Falkensammer ML, Gobet R, Stauffer UG, Weber DM (2008). To cohen and forget? Evaluation of postoperative imaging studies after transtrigonal ureteric reimplantation for vesicoureteric reflux in children. *Urologia Internationalis*.

[20] Charbonneau SG, Tackett LD, Gray EH, Caesar RE, Caldamone AA (2005). Is long-term sonographic followup necessary after uncomplicated ureteral reimplantation in children?. *Journal of Urology*.

[21] Bomalaski MD, Ritchey ML, Bloom DA (1997). What imaging studies are necessary to determine outcome after ureteroneocystostomy?. *Journal of Urology*.

[22] Gearhart JP, Gearhart JP, Rink RC, Mouriquand PDE (2011). The bladder exstrophy-epispadias-cloacal exstrophy complex. *Pediatric Urology*.

[23] Mouriquand PDE, Bubanj T, Feyaerts A (2003). Long-term results of bladder neck reconstruction for incontinence in children with classical bladder exstrophy or incontinent epispadias. *BJU International*.

[24] Gearhart JP (1995). Bladder exstrophy and urinary continence: long term outcomes. *Pediatric Surgery*.

[25] Elder JS (1990). Periurethral and puboprostatic sling repair for incontinence in patients with myelodysplasia. *Journal of Urology*.

[26] Pippi Salle JL (2001). Pippi Salle onlay procedure. *Atlas of Urologic Clinics of North America*.

[27] Hoebeke P, De Kuyper P, Goeminne H, Van Laecke E, Everaert K (2000). Bladder neck closure for treating pediatric incontinence. *European Urology*.

[28] Mitrofanoff P (1980). Cystostomie continente trans-appendiculaire dans le traitement des vessies neurologiques. *Chiropractic Pediatrics*.

[29] Politano VA (1978). Periurethral teflon injection for urinary incontinence. *Urologic Clinics of North America*.

[30] Vorstman B, Lockhart J, Kaufman MR, Politano V (1985). Polytetrafluoroethylene injection for urinary incontinence in children. *Journal of Urology*.

[31] Halachmi S, Farhat W, Metcalfe P, Bagli DJ, McLorie GA, Khoury AE (2004). Efficacy of polydimethylsiloxane injection to the bladder neck and leaking diverting stoma for urinary continence. *Journal of Urology*.

[32] Godbole P, Bryant R, MacKinnon AE, Roberts JP (2003). Endourethral injection of bulking agents for urinary incontinence in children. *BJU International*.

[33] Caione P, Capozza N (2002). Endoscopic treatment of urinary incontinence in pediatric patients: 2-Year experience with dextranomer/hyaluronic acid copolymer. *Journal of Urology*.

[34] Lottmann HB, Margaryan M, Lortat-Jacob S, Bernuy M, Läckgren G (2006). Long-term effects of dextranomer endoscopic injections for the treatment of urinary incontinence: an update of a prospective study of 61 patients. *Journal of Urology*.

[35] Guys JM, Fakhro A, Louis-Borrione C, Prost J, Hautier A (2001). Endoscopic treatment of urinary incontinence: long-term evaluation of the results. *Journal of Urology*.

[36] Dyer L, Franco I, Firlit CF, Reda EF, Levitt SB, Palmer LS (2007). Endoscopic injection of bulking agents in children with incontinence: dextranomer/hyaluronic acid copolymer versus polytetrafluoroethylene. *Journal of Urology*.

[37] Burki T, Hamid R, Ransley PG, Mushtaq I, Duffy PG (2006). Injectable polydimethylsiloxane for treating incontinence in children with the exstrophy-epispadias complex: long-term results. *BJU International*.

